# The Protective Antibodies Induced by a Novel Epitope of Human TNF-α Could Suppress the Development of Collagen-Induced Arthritis

**DOI:** 10.1371/journal.pone.0008920

**Published:** 2010-01-27

**Authors:** Jie Dong, Yaping Gao, Yu Liu, Jinxia Shi, Jiannan Feng, Zhanguo Li, Heping Pan, Yanning Xue, Chuan Liu, Beifen Shen, Ningsheng Shao, Guang Yang

**Affiliations:** 1 Beijing Institute of Basic Medical Sciences, Beijing, China; 2 Department of Rheumatology and Immunology, People's Hospital, Peking University, Beijing, China; Ohio State University, United States of America

## Abstract

Tumor necrosis factor alpha (TNF-α) is a major inflammatory mediator that exhibits actions leading to tissue destruction and hampering recovery from damage. At present, two antibodies against human TNF-α (hTNF-α) are available, which are widely used for the clinic treatment of certain inflammatory diseases. This work was undertaken to identify a novel functional epitope of hTNF-α. We performed screening peptide library against anti-hTNF-α antibodies, ELISA and competitive ELISA to obtain the epitope of hTNF-α. The key residues of the epitope were identified by means of combinatorial alanine scanning and site-specific mutagenesis. The N terminus (80–91 aa) of hTNF-α proved to be a novel epitope (YG1). The two amino acids of YG1, proline and valine, were identified as the key residues, which were important for hTNF-α biological function. Furthermore, the function of the epitope was addressed on an animal model of collagen-induced arthritis (CIA). CIA could be suppressed in an animal model by prevaccination with the derivative peptides of YG1. The antibodies of YG1 could also inhibit the cytotoxicity of hTNF-α. These results demonstrate that YG1 is a novel epitope associated with the biological function of hTNF-α and the antibodies against YG1 can inhibit the development of CIA in animal model, so it would be a potential target of new therapeutic antibodies.

## Introduction

Tumor necrosis factor alpha (TNF-α) is an inflammatory cytokine primarily secreted by the macrophages/monocytes in response to a variety of stresses that interfere drastically with the growth, differentiation, and death of both immune and nonimmune cell types, and simultaneously stimulate a series of other proinflammatory mediators [1]. TNF-α is initially synthesized as cell surface-bound precursor transmembrane TNF (tmTNF, a homotrimer of 26-kDa monomers), then cleaved to the soluble-form TNF-α (sTNF, a monomer of 17 kDa) by TNF-alpha-converting enzyme (TACE). Both sTNF and tmTNF ligands interact with either of 2 distinct receptors—TNF receptor 1 (TNFR1, p55, CD120a) and TNF receptor 2 (TNFR2, p75, CD120b)—on a wide variety of cell types to mediate their biological functions [2], [3].

Rheumatoid arthritis (RA) is a systemic, progressive, inflammatory, autoimmune disorder that targets primarily the synovial tissues and leads to destruction of cartilage and ultimately bone. The conventional disease-modifying anti-rheumatic drugs (DMARDs) can efficiently improve sign and symptoms and increase functional ability. However, they can do little on halting progressive joint damage. The breakthrough in development of biological agents for the treatment of RA was to target the immune system, which was ascribed to new insight into the major biological function of TNF-α in joint inflammation and destruction. The three currently available TNF antagonists, adalimumab, a fully human monoclonal antibody; infliximab, a chimeric monoclonal antibody; and etanercept, a soluble receptor construct, have changed the course and face of rheumatoid arthritis and consequently the outcomes for patients and society, especially in combination with methotrexate [3]–[5]. Although the potential risks of infection, lymphoma, solid tumor and congestive heart failure would increase when these TNF-α antagonists are used in clinical applications[6]–[8], efficacy and safety of the treatments are validated in large clinical cases, especially in those which do not respond to traditional treatments[9], [10].

In this study, we identified a novel precise epitope of hTNF-α, which could not be recognized by those commercial antibodies. Moreover, we found that antibodies induced by the derivative peptides of this epitope could suppress the cytotoxicity of hTNF-α and the development of collagen-induced arthritis (CIA) in an animal model of RA. Thus, this epitope is a potential new target for the development of TNF-α blockade agents.

## Results

### Identification of the hTNF-α Mimotope and Epitope

**Figure 1:**
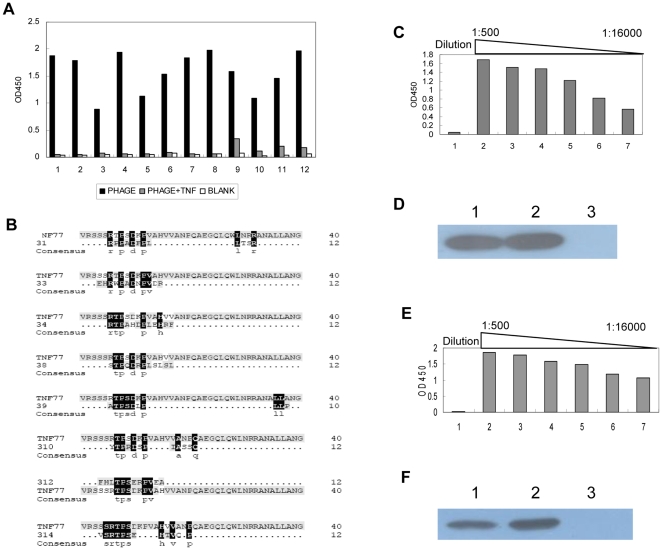
Identification of the mimotope and epitope of hTNF-α. **A:** Specific phage clones binding to rhTNF-α antibodies were selected by ELISA and competitive ELISA. 1–12 referred to different phage clones. White bars represent negative control, black bars represent screened phage clones (1×10^9^) alone, and gray bars represent screened phage clones (1×10^9^) plus rhTNF-α protein (5 µg). **B:** Sequence similarity between binding peptides and rhTNF-α. 31, 33, 34, 38, 39, 310, 312 and 314 represent different binding peptides. All the selected binding peptides were homologous to the region 80–91aa of rhTNF-α. **C:** Development of anti-rhTNF-α antibodies using KLH-312.The interaction between rhTNF-α and sera was tested by ELISA. Results are expressed as OD at 450 nm. Group1: the control group sera immunized with KLH diluted as 1∶500; group2–7: sera immunized with KLH-312 were diluted from 1∶500–1∶16000. The experiment was performed twice. **D:** The interaction between rhTNF-α and sera was tested by Western Blot. The rhTNF-α protein was applied to SDS-PAGE and transferred to the membrane. And the antisera of peptides were added, the antisera of hTNF-α as the positive control. And the antisera of KLH were taken as the negative control. The antisera are diluted as 1∶500. Lane1: antisera against rhTNF-α; Lane2: antisera against KLH-312; Lane3: antisera against KLH. **E:** Development of anti rhTNF-α antibodies using KLH-YG1, The interaction between rhTNF-α and sera was tested by ELISA. Results are expressed as OD value at 450 nm. Group1: control group sera immunized with KLH diluted as 1∶500; Group2–7: sera immunized with KLH-YG1 were diluted from 1∶500–1∶16000. The experiment was performed twice. **F:** The interaction between rhTNF-α and sera was tested by Western Blot. The method was described as figure1D. The antisera are diluted as 1∶500. Lane1: antisera against rhTNF-α; Lane2: antisera against KLH-YG1; Lane3: antisera against KLH.

Polyclonal antibodies against rhTNF-α were prepared and purified as described in “Materials and methods”. The phage clones were isolated by incubating the 12-mer linear random peptide library with the antibodies after 3 rounds of bioscreening. Competitive enzyme-linked immunosorbent assay (ELISA) was employed to select 12 positive phage clones(**Figure 1A**), and the selected clones were then sequenced. The sequencing result showed that eight different sequences were captured, then the sequence identity between these binding peptides and hTNF-α was analyzed. We found that the sequences of all the selected peptides were similar to the sequence of amino acids 80–91 in hTNF-α(**Figure 1B**). There were 5 clones with the same amino acid sequence FHLTPSERPVEA in the selected 12 positive clones. This sequence was with high similarity to the natural residues 80–91 of human TNF, designated as 312. The peptide 312 was selected, synthesized, and conjugated to keyhole limpet hemocyanin (KLH). Antisera against the conjugate were prepared and were found to exhibit reactivity toward rhTNF-α by performing ELISA and western blotting (**Figure 1C, 1D**). This peptide was predicted to be the mimotope of hTNF-α, and the homologous region in hTNF-α was assessed as a potential epitope. Next, the peptide that corresponded to the 80–91 residues of hTNF-α was synthesized, conjugated to KLH, and used for vaccination. As expected, the results of ELISA and Western Blot revealed that the antisera could react with rhTNF-α (**Figure 1E, 1F**). Our results suggested that this epitope should be a linear epitope of rhTNF-α.

### No Cross-Reactivity between the Epitope and the Commercial Antibodies

**Figure 2:**
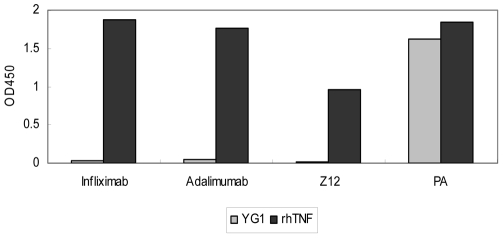
The interaction between different antibodies and YG1. The synthesized peptide according to YG1 and rhTNF-α were individually coated. Different antibodies were added, Infliximab, Adalimumab and Z12 referred to three monoclonal antibodies, while PA was the polyclonal antibodies against rhTNF-α. The results were expressed as OD value at 450 nm. The experiment was performed twice.

As described above, the commercial antibodies infliximab, which is a chimeric (75% human and 25% mouse peptide sequences) monoclonal antibody against hTNF-α and adalimumab, which is the first fully human antibody (100% human peptide sequences) against hTNF-α have been available thus far. To analyze whether the epitope identified in our study could react to these antibodies, the synthetic peptide (10 µg/well) and rhTNF-α (2 µg/well) were individually coated over the wells of microtiter plates, respectively, following which the 100 µl monoclonal antibodies (infliximab, adalimumab, and Z12 at the concentration of 1 µg/ml) or 100 µl polyclonal anti-hTNF-α antibodies (PA) with the same concentration with monoclonal antibodies were added. The results of ELISA revealed that all of the antibodies could bind hTNFα, while contrasted with PA, the commercial Abs couldn't bind the peptide (**Figure 2**). This result confirmed that the 80–91 residue of hTNF-α was a novel epitope, and we designated it as YG1.

### Inhibition of the rhTNF-α Cytotoxicity by Antibodies against YG1

**Figure 3:**
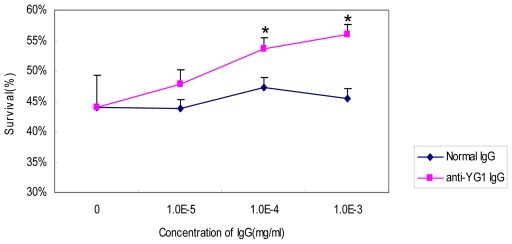
anti-YG1 Ab could inhibit the cytotoxicity of rhTNF-α. Various concentrations of antibodies against to YG1 and control antibodies were added to inhibit the cytotoxicity of rhTNF-α. The cytotoxicity was measured by detecting the amounts of cells by MTT. Values represent the mean ± SD for 3 independent tests. * represent P<0.001(anti-YG compared with normal Ab).

Anti-YG1 Ab was purified by YG1 conjugated Sulfolink coupling resin (Pierce) according to the manufacture's instruction. The rhTNF-α (final concentration 5 pg/ml), with different dilution of anti-YG1 antibodies, was added to each well. The actinomycin D was added with final concentration 1 µg/ml. The plates were incubated at 37°C for 18–24 h incubation in 5% CO_2_. The cell viability was measured by MTT assay. Absorbance was measured at a wavelength of 595 nm to calculate the percentage of viable cells. The result showed that anti-YG1 antibodies could statistically inhibit cytotoxicity of hTNF-α and increase survival of the L929 cells in a dose dependent manner, compared with the control of normal antibodies (**Figure 3**).

### YG1 Could Suppress the Development of CIA in an Animal Model of RA

RA is a chronic and inflammatory disorder characterized by polyarthritis with progressive joint erosion and dysfunction. Previous studies suggest that: (1) TNF-α is expressed at high levels in the inflamed synovium, particularly at the cartilage-pannus junction of RA patients [11]–[13]; and (2) TNF-α inhibitors (eg. infliximib, adalimumab, and etanercept-a recombinant hTNF receptor (p75)-Fc fusion protein) have been used in the RA treatment in clinical practice [14], [15].

**Figure 4:**
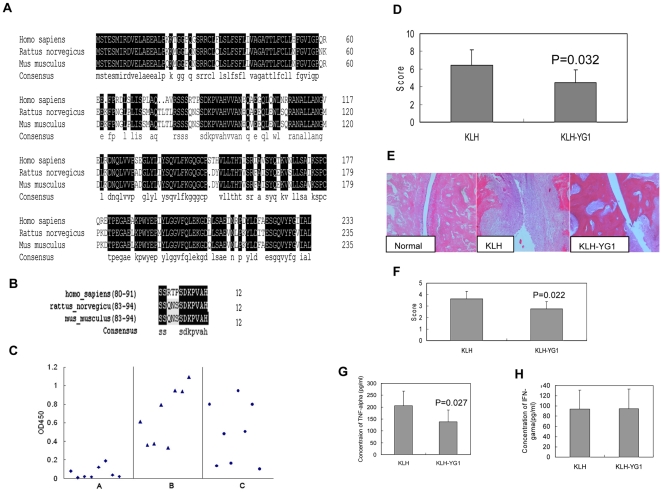
YG1 could suppress the development of collagen-induced arthritis in the Rheumatoid arthritis animal model. **A:** The homology analysis of TNF-α in human, mice and rats. **B:** The homology analysis of the region of TNF-α according to YG1 in human, mice and rats. **C:** The detection of antisera against rhTNF-α in rats. Rats in Group A were prevaccinated with KLH, while Rats in Group B and C were prevaccinated with KLH-YG1. The sera were diluted as 1∶100, and the interaction between rhTNF-α and sera was tested by ELISA. **D:** Arthritis score. KLH represents the rats of Group A immunizied with CII, and KLH-YG1 represents the rats of Group B immunizied with CII. **E:** Histologic analysis of ankle joints in CIA rats. Normal represents the normal rats, KLH represents the rats Group A immunizied with CII, and KLH-YG1 represents Group B immunizied with CII (Original magnification ×10). **F:** Histologic score of ankle joints in CIA rats. KLH represents the rats Group A immunizied with CII, and KLH-YG1 represents Group B immunizied with CII. **G, H:** Detection the concentration of different cytokines in blood. The production of TNF-α and IFN-γ was analyzed in the different groups.

An animal model of CIA, which is a type of polyarthritis with many histopathologic features similar to those of RA, was generated. The TNF-α and the region according to YG1 were highly conserved in human, mice and rats (**Figure 4A, 4B**). We attempted to test the function of the newly identified epitope in such an animal model. Twenty-four rats were separated into 3 groups (A, B, and C, 8 rats each). The rats in group A were immunized with KLH alone, while those in group B and C were prevaccinated with KLH-YG1, as described in “Materials and methods.” The interaction between anti-YG1 sera and rhTNF-α was tested by performing ELISA. The results revealed that YG1 could induce the production of specific antisera against hTNF-α (**Figure 4C**) in rats. Further, the animal in group A and B received 2 immunizations with bovine collagen type II (CII), and rats of group C served as controls. From day 12 to 14 after the primary immunization, swelling was noted in the joints of the 16 rats in groups A and B. The arthritis scores were significantly lower in the KLH-YG1-treated rats than in the KLH-treated rats (**Figure 4D**). The group C rats showed no loss of body weight and did not develop any skin lesions. These results indicated that YG1 treatment could suppress the development of CIA. Histologic examination of the joints revealed severe articular cartilage erosion, bone erosion, massive inflammatory cell infiltration, and obvious pannus formation in the control group. Moderate or mild synoviocyte hyperplasia and articular cartilage erosion were noted in the YG1-KLH-treated rats, and the degree of arthritis was significantly lower in these rats than in the KLH-treated rats (2.75±1.12 versus 3.18±0.85, P<0.05) (**Figure 4E, 4F**). We also assessed the cytokine levels (TNF-α and IFN-γ) in the blood of the rats in each group. The results indicated that TNF-α production was significantly reduced in group B as compared to group A (**Figure 4G**); however, no difference was observed between the 2 groups with regard to the blood level of IFN-γ (**Figure 4H**).

### Identification of the Key Amino Acids in YG1

**Figure 5:**
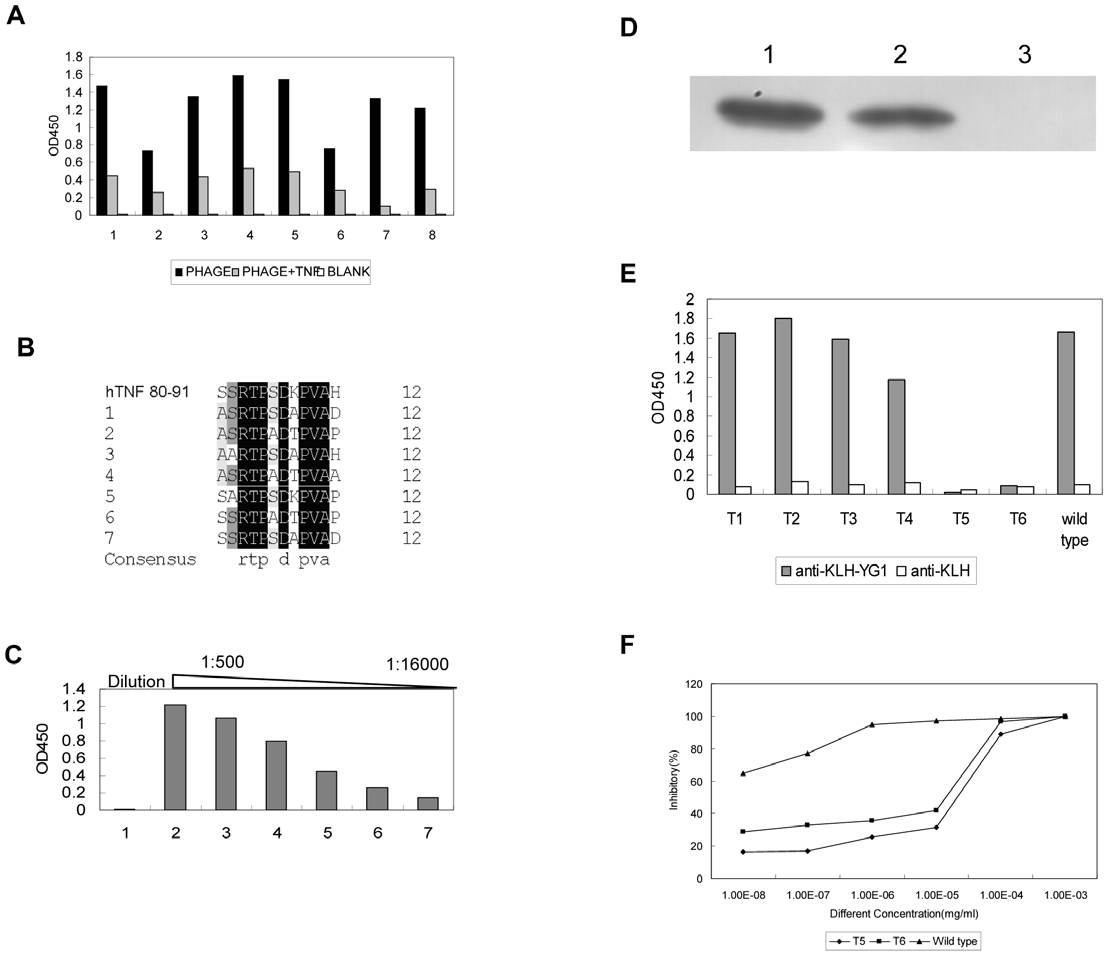
Identification of key amino acid in YG1. **A:** Specific phage clones binding to rhTNF-α antibodies were selected by ELISA and competitive ELISA. 1–8 referred to different phage clones. White bars represent negative control, black bars represent screened phage clones (1×10^9^) alone, and gray bars represent screened phage clones (1×10^9^) plus rhTNF-α protein (5 µg). **B:** Sequence similarity between binding peptides obtained by Alanine Scanning and the N terminus of rhTNF-α. The alignment of different sequences was proceeded by the software DNAMAN. 1–7 referred to different binding peptides sequences. The conserved sequence is XXRTPXDXPVAX (X means any amino acid). **C:** Development of anti-rhTNF-α antibodies using KLH-AP. The interaction between rhTNF-α and sera was tested by ELISA. Results are expressed as OD value at 450 nm. Group1 represents control group sera immunized with KLH, Group2–7 represent sera immunized with KLH-AP were diluted from 1∶500–1∶16000. **D:** The interaction between rhTNF-α and sera was tested by Western blot. Different antisera was diluted as 1∶500. Lane1 represents antisera against rhTNF-α, and Lane2 represents antisera against KLH-AP, Lane3 represents antisera against KLH. **E:** The interaction between different rhTNF-α mutants and sera immunized with KLH-YG1 was tested by ELISA. The result of ELSIA was expressed as OD at 450 nm. T1, R82A; T2, T83A; T3, P84A; T4, D86A; T5, P88A and T6, V89A. The experiment was performed twice. **F:** Detection of the cytotoxicity induced by rhTNF-α and mutants. Serial dilutions of rhTNF-α and mutants (T5, T6) were incubated with L929 cells. The cytotoxicity was measured by detecting the amounts of cells by MTT (the triangle bar: wild type, the diamond bar:T5, the square bar: T6). The experiment was performed twice.

Further, we attempted to identify the key amino acids in YG1 by constructing an alanine-scanning library. First, a phage-displayed 12-mer peptide library, wherein the residues are ideally allowed to vary only as one of the wild-type amino acids or alanine, was constructed on the basis of the YG1 sequence (**Table 1**), although the nature of the genetic code necessitates 2 other amino acids substitutions for some residues. Twenty clones were randomly selected and sequenced; the results revealed that the distribution of nucleotides at each position was almost identical with the expected frequency (data not shown). The original transformants were 4.0×10^4^, and the theoretical deviation of the library was 1.6×10^4^, so the constructed library should consist of all mutagenic oligonucleotide. The peptides in the library that were specifically bound to the anti-TNF-α antibodies were subsequently selected and sequenced (**Figure 5A**). With sequence analysis, 7 conserved amino acids in the selected sequences were identified (**Figure 5B**). The sequence alignment between the mimotope and rhTNF-α (**Figure 1B**) suggested that alanine might not have been the key residue in the epitope. The fact that alanine was identified as one of the conserved amino acids may be a limitation of this method because alanine in YG1 could not be replaced in the alanine-scanning library. An artificial peptide (AP), wherein the residues other than the key amino acids were replaced with alanine, was synthesized and coupled with KLH. Mice were then vaccinated with this complex for the production of polyclonal antibodies. The results of ELISA and Western Blot revealed that the antisera could specifically bind to rhTNF-α (**Figure 5C, 5D**). Thus, we concluded that the selected residues were sufficient to induce the production of anti-TNF-α antibodies.

**Table 1:** The shotgun scanning code.

Amino acid	Shotgun codon*	Shotgun substitutions
S	KCT	A/S
R	RSG	A/G/T/R
T	RCT	A/T
P	SCT	A/P
S	KCT	A/S
D	GMT	A/D
K	RMG	A/E/T/K
P	SCT	A/P
V	GYT	A/V
A	GCT	A
H	SMT	A/D/P/H

For each amino acid, the appropriate shotgun codon ideally encodes only the wild-type amino acid or alanine, but the nature of the genetic code necessitates the occurrence of 2 other amino acids for some shotgun substitutions. Single-letter amino acid and nucleotide abbreviations are used.

* DNA degeneracies are represented by the IUB code (K = G/T, M = A/C, N = A/C/G/T, R = A/G, S = G/C, W = A/T, Y = C/T).

On the basis of the key residues, 6 rhTNF-α mutants (T1, R82A; T2, T83A; T3, P84A; T4, D86A; T5, P88A; and T6, V89A) were expressed and purified, as described in “Materials and methods”. We then tested the binding between the mutants and the anti-YG1 sera. The results of ELISA demonstrated that the anti-YG1 sera could not recognize the two rhTNF-α mutants P88A and V89A (**Figure 5E**). These results suggested that the residues Proline and Valine were the most critical amino acids in YG1. Further, we tested the activities of the abovementioned mutants by assessing the TNF-α-induced cytotoxicity. The results revealed that the two mutants P88A and V89A was significantly less cytotoxic than wild-type rhTNF-α (**Figure 5F**) and that the two abovementioned key amino acids in YG1 were also important for the biological functions of TNF-α.

## Discussion

One of the critical findings is that proinflammatory cytokines such as TNF-α were present in the synovium and plasma of patients with RA. It has thrown new light on the attempts to develop RA therapies since 1980s [11]–[13]. Despite of the potential adverse effects and limitations, the licensed anti-TNF-α monoclonal Abs, adalimumab and infliximab, have proved to be remarkably successful as an injectable protein-based therapy in clinical treatment of immune-mediated inflammatory diseases (IMID), such as rheumatoid arthritis (RA), Crohn's disease, inflammatory bowel disease (IBD), psoriatic arthritis (PsA), and ankylosing spondylitis(AS) [16]. Intriguingly, the precise mechanisms, by which infliximab and adalimumab work, are in part different, such as effects of TNF-α blockade on reverse (outside to inside) intracellular signaling cascade, infiltrating cells, epithelial cells' barrier function [17]. It probably explains why there are some differences in the indications of the two monoclonal Abs. For example, infliximab approved to be applied on ulcerative colitis patients, while adalimumab is lack of efficacy in such disease. It's also comprehensible that some RA patients intolerant of one of the Abs could switch to the other, which would be safe and effective [18]. Those results suggested the two Abs would bind the different sites of TNF-α. So we wonder whether there are some other epitopes of hTNF-α for developing novel therapeutic antibodies.

In this work, we tried to obtain the epitopes by biopanning phage-displayed peptide library against anti-hTNF-α polyclonal Abs. The positive phage clones identified by ELISA assay exhibited 8 different sequences, in which there was one similar sequence embedded in hTNF-α(80-91aa). The antisera raised against the selected peptide 312 or the peptide YG1 of hTNF-α(80–91aa) had the specific reactivity with rhTNF-α, which suggested that both the mimotopes of hTNF-α and the corresponding epitope exhibited similar immunogenicity. The key amino acids of an epitope usually comprise 3∼5 residues, so we identified the key ones in YG1 by means of alanine-scanning and site-directed mutation. Proline and Valine proved to be the most important amino acids in YG1.

It was found that the epitope peptide YG1 could not be recognized by the marketed Abs, so it suggested that YG1 was a novel epitope of hTNF-α. The biological function of YG1 was elucidated in rat model of CIA. The progress of CIA is associated with the level of TNF-α, while the rats vaccinated with YG1 showed less severity of inflammatory reaction, contrasted with the control. We also indentified that the antibodies against YG1 could efficiently inhibit the cell toxicity induced by hTNF-α. The epitope YG1 is not located at the receptor-binding site (150–155 residues) of hTNF-α [19], so the antibodies against YG1 might not directly inhibit the binding hTNF-α and receptor. The mechanism how the antibodies of YG1 inhibit the function of hTNF-α may be different from the present antibodies and still remains unclear. We tried to construct the theoretical 3-D trimer structure of TNF and 2 mutants (P88A and V89A) on the basis of the 3-D crystal structure of TNF (PDB code: 1tnf). The preliminary data showed that it was more difficult for T5 and T6 to form trimer than for wild types (data not shown). The further mechanism is being addressed in our lab. However, our results suggested that there should be other sites (including YG1) at hTNF-α for novel antagonists development.

Our work also reveals that combination of phage display and alanine-scanning is an efficient way to identify the novel precise epitopes in a disease-related molecule. It will be useful for development and optimization of corresponding targeting therapy.

## Materials and Methods

### Ethics Statement

All animal experimental protocols of the study are in accordance with the national guidelines for the use of animals in scientific research. It's also approved by Animal Care and Use Committee of the Peking University People's Hospital.

### Antibodies

Infliximab, adalimumab, and Z12 were kindly provided by Professor Yan Li (Beijing Institute of Basic Medical Sciences). Z12 is a monoclonal antibody developed in the laboratory of Professor Yan Li and contains a distinct epitope; its structure is comparable to those of the 2 commercial antibodies (unpublished data).

### Phage Display Peptide Library, Bacterial Strains, and Animals

The Ph.D.-12™ phage display peptide library kit was purchased from New England Biolabs (Beverly, MA, USA). The complexity of the library was 1.9×10^9^, and the titer was 1.5×10^13^ pfu/µl. The *E. coli* strain ER2537 was used as the host strain for the phage library. The Ph.D.™ Peptide Display Cloning System was also purchased from New England Biolabs. The *E. coli* strain BL21 was purchased from Novagen. Female New Zealand white rabbits and outbred male BALB/c mice (weight, 20–25 g) were provided by Beijing Research Center of Animals. Inbred female Lewis rats were purchased from Laboratory Animal Resource Center of Peking University Medical Science Center. The rats were housed in a specific pathogen-free environment and were provided standard rodent chow diet ad libitum.

### Production and Purification of Polyclonal Anti-Recombinant Human TNF-α Antibodies

Purified recombinant human TNF-α (rhTNF-α) was produced as described previously [20]. Female New Zealand White rabbits were first immunized by subcutaneously injecting them with 1 ml of the immunogen (1 mg of rhTNF-α in phosphate-buffered saline (PBS) mixed with complete Freund's adjuvant (Sigma)). Subsequent booster injections, i.e., 1 mg of rhTNF-α in PBS emulsified in incomplete Freund's adjuvant (Sigma), were administered at 3 and 6 weeks after the primary immunization. At the eighth week, the sera were collected and the antibody titers were determined by ELISA. Anti-rhTNF-α antibodies were purified on an immuno-affinity column which was prepared by conjugating purified rhTNF-α to carboxylated acrylonitrile butadiene (CNBr)-activated Sepharose™ 4B resin (Amersham Biosciences), according to the manufacturer's instructions.

### Selection of Peptides Binding to rhTNF-α Antibodies by Phage Display

The selection procedure was in accordance with the protocol provided with the Ph.D.-12 peptide library kit (New England Biolabs). For each selection cycle, 1×10^11^ phages were applied to a 96-well plate precoated with anti-rhTNF-α antibodies (10 µg/well). The level of specific phage enrichment was calculated as the input-output ratio, as described previously [21]. After 3 rounds of biopanning, the positive phage clones were selected and sequenced.

### ELISA

The specific binding of the positive phage clones to the polyclonal anti-rhTNF-α antibodies was tested by performing ELISA. In brief, the 96-well plates (Nunc) were coated with anti-rhTNF-α polyclonal antibodies (10 µg/well) overnight at 4°C. The unbound antibodies were discarded, and the wells were blocked with 3% bovine serum albumin in PBS at 37°C for 1 h. The selected phage clones (1×10^9^ pfu/well) were added to the wells in duplicate, and the plates were incubated at 37°C for 2 h. The plates were then washed 5 times with PBS-0.05% Tween-20, following which a horseradish peroxidase (HRP)-conjugated anti-M13 polyclonal antibody (1∶1000), (Amersham Biosciences) was added, and the plates were incubated at 37°C for 1 h. After the plates were washed as described above, the bound antibodies were detected using 3,3′,5,5′-tetramethylbenzidine dihydrochloride (Sigma, St. Louis, USA) as the substrate, and the color intensity was determined spectrophotometrically at 450 nm.

### Western Blot

rhTNF-α protein (2 µg) was subjected to 15% SDS-PAGE, then blotted onto Hybond-ECL nitrocellulose membrane (Amersham Biosciences) for 30 min at 20 V. After blocking for 2 h at 37°C in 5% non-fat milk, the membrane was probed with various antisera (1∶500), diluted in blocking buffer for 1 hr at room temperature, and washed twice with 0.5% PBST. Then the membrane was incubated in a goat anti-rabbit HRP-conjugated secondary antibody (1∶2,000), diluted in blocking buffer, and washed four times with 0.5% PBST. The binding of rhTNF-α with corresponding antibodies was detected by the enhanced chemiluminescence (ECL) Western blotting detection system.

### Competitive ELISA

Various concentrations of the rhTNF-α protein along with 1×10^9^ phage particles were added into each well of the 96-well plates (Nunc); the wells were precoated with anti-rhTNF-α antibodies. The bound phages were detected as described above using HRP-conjugated anti-M13 polyclonal antibody.

### Induction of CIA and Evaluation of Its Clinical Severity

Five-week-old Lewis rats were prevaccinated with KLH-YG1 or KLH. The rats were first immunized by subcutaneously injecting them with 0.5 ml of the immunogen (0.5 mg of KLH-YG1 or KLH in PBS mixed with complete Freund's adjuvant). Subsequent booster injections, comprising 0.25 mg of KLH-YG1 or KLH in PBS emulsified in incomplete Freund's adjuvant (IFA), were administered 1 week after the primary immunization. The prevaccinated rats were then immunized with bovine CII (Sigma) as described previously [22], [23]. Briefly, bovine CII was dissolved in 0.1 M acetic acid at a concentration of 4 mg/ml and emulsified in an equal volume of IFA (Sigma). The rats were immunized intradermally by injecting them at the base of the tail with 150 µl of the emulsion (containing 300 µg of CII) on days 0 and 7. The incidence and severity of induced arthritis were determined by assessing the weight loss and scoring each paw on the basis of the degree of swelling, erythema, and deformity in the joints [11]. The arthritic lesions were scored on a scale of 0–4, where a score of 0 corresponded to no change; 1, swelling and erythema of the digit; 2, mild swelling and erythema of the limb; 3, gross swelling and erythema of the limb; and 4, gross deformity and inability to use the limb. The arthritis score recorded for each rat was the sum of the scores of the individual paws; the maximum score was 16.

### Histopathological Analysis

The hind paws of the rats in different groups were dissected, fixed in 10% neutral buffered formalin for 48 h, and decalcified in 10% (w/v) ethylenediaminetetraacetic acid disodium salt (EDTA-2Na) until the bones were pliable. The tissues were then dehydrated in an alcohol gradient, embedded in paraffin, cut into 4-µm thick sections, and stained with hematoxylin and eosin. The sections were analyzed microscopically to observe the degree of inflammation and cartilage and bone destruction, according to a previously reported method [24]. The following scale was used to assess this degree: 0, normal synovium; 1, synovial membrane hypertrophy and cell infiltration; 2, pannus and cartilage erosion; 3, major erosion of the cartilage and subchondral bone; and 4, loss of joint integrity and ankylosis.

### Analysis of Cytokine Production

Blood samples were collected on day 35 after the primary immunization. The serum levels of TNF-α (Dakewei Biotech Company Ltd., China) and IFN-γ (Jingmei, China) were measured using commercially available ELISA kits according to the manufacturer's instructions.

### Construction of the Alanine-Scanning Library

The libraries were constructed using the M13KE gIII cloning system (NEB). A template oligonucleotide with the sequence 5′-CATGTTTCGGCCGAAKSAGCAR-CAGSCKYAKCAGMAGSAGYCSYAGMAGMAGAGTGAGAATAGAAAGGTACCCGGG-3′ (DNA degeneracies are represented by the International Union of Biochemistry (IUB) code (K, G/T; M, A/C; N, A/C/G/T; R, A/G; S, G/C; W, A/T; Y, C/T)), which contained the fragment encoding the insert sequence of hTNF-α (80–91) or Ala-mutated sequences, was annealed to the extension primer. This template was then extended using DNA polymerase, large (Klenow) fragment (NEB) and converted into the double-stranded form. The double-stranded DNA was digested with *Acc65* I and *Eag* I to release an internal 60-bp fragment. This fragment was purified by 12% polyacrylamide gel electrophoresis in Tris–borate–EDTA (TBE) and was eluted with a buffer containing 0.5 M ammonium acetate (NH_4_Ac), 10 mM magnesium acetate (Mg (Ac)_2_), and 1 mM EDTA. The eluate was purified and concentrated by ethanol precipitation. The purified fragment was ligated into the M13KE vector that was double-digested with *Acc65* I and *Eag* I. The ligation product was transfected into competent ER2537 cells by 10 sessions of electroporation. The transformation mixture was grown in a 200 ml suspension of cells early-log phase in Luria-Bertani (LB) medium for 4.5 h. The phage was purified according to the manufacturer's instructions. Meanwhile, 20 original transformants were randomly selected for sequencing. Furthermore, the constructed alanine-scanning library was screened with anti-rhTNF-α antibodies for three rounds.

### Expression and Purification of Recombinant hTNF-α and the Site-Specific Mutants

rhTNF-α and its mutants were expressed in *E. coli* by using the conventional methods. In brief, the genes coding rhTNF-α and its mutants were amplified by polymerase chain reaction (PCR) using the template DNA pUC-rhTNF-α vector (stored in our lab) with the individual forward primers(**wild type(WT):** GGGAATTCCATATGGTACGTTCTTCTTCTCGTACTCCGAGTGACAAGCCTGTAGCCCATGTTGTAG, **R82A (T1**): GGGAATTCCATATGGTACGTTCTTCTTCT *gc*TACTCCGAGTGACAAGCCTGTAGCCCATGTTGTAG, **T83A(T2**):GGGAAT TCCATATGGTACGTTCTTCTTCTCGT*g*CTCCGAGTGACAAGCCTGTAGCCCATGTTGTAG, **P84A (T3**): GGGAATTCCATATGGTACGTTCTTCTTCTCGTACT*g*CGAGTGACAAGCCTGTAGCCCATGTTGTAG, **D86A (T4**):GGGAATTCCA TATGGTACGTTCTTCTTCTCGTACTCCGAGTG*c*CAAGCCTGTAGCCCATGTTGTAG, **P88A (T5):** GGGAATTCCATATGGTACGTTCTTCTTCTCGTACTCCGA GTGACAAG*g*CTGTAGCCCATGTTGTAG, **V89A (T6):** GGGAATTCCATATGGT ACGTTCTTCTTCTCGTACTCCGAGTGACAAGCCTG*c*AGCCCATGTTGTAG, the lowercased and italicized letters indicate the mutation sites, and the underlined sequences indicate *Nde*I recognition sites) and the same reverse primer(CCGCTCGAGTCACAGGG CAATGATCCCAA, the underlined sequences indicate *Xho*I recognition sites). The amplified fragments were digested with *Nde*I and *Xho*I and inserted into the corresponding sites in the pET-28a vector (Novagen) sequence. Recombinant protein expression was induced by adding isopropyl β-d-thiogalactopyranoside (IPTG) (0.1 mM) to the growing culture at an optical density of 0.4 at 600 nm (OD600). The recombinant protein was purified on a Ni Sepharose™ 6 Fast Flow column (Amersham Biosciences), according to the manufacturer's instructions.

### Detection of the Cytotoxicity Induced by rhTNF-α and Its Mutants

In brief, L929 (4×10^4^ cells/ml, 100 µl/well) were seeded in 96-well microtiter plates (Costar). They were then cultured overnight at 37°C in complete Dulbecco's modified Eagle's medium (DMEM) (Gibco) containing 10% fetal bovine serum (Hyclone), in the presence of 5% CO_2_. The rhTNF-α and mutants were serially diluted. Further, 20 µl of each dilution along with 5 µl actinomycin D (25 µg/ml) was added to each well, and the plates were incubated at 37°C for 18–24 h incubation in 5% CO_2_. The cell viability was measured by performing a standard 3-(4,5- dimethylthiazol-2-yl)-2,5-diphenyltetrazolium bromide assay (MTT). Absorbance was measured at a wavelength of 595 nm to calculate the percentage of viable cells.
